# Towards Human Sensory Augmentation: A Cognitive Neuroscience Framework for Evaluating Integration of New Signals within Perception, Brain Representations, and Subjective Experience

**DOI:** 10.1007/s41133-024-00075-7

**Published:** 2024-10-28

**Authors:** Marko Nardini, Meike Scheller, Melissa Ramsay, Olaf Kristiansen, Chris Allen

**Affiliations:** Department of Psychology, Durham University, Durham, UK

**Keywords:** Human-centered computing → Human computer interaction (HCI); Visualization; Accessibility, Sensory substitution, Sensory augmentation, Assistive augmentation, Augmented reality, Wearable computing, Perception, Cognitive neuroscience

## Abstract

New wearable devices and technologies provide unprecedented scope to augment or substitute human perceptual abilities. However, the flexibility to reorganize brain processing to use novel sensory signals during early sensitive periods in infancy is much less evident at later ages, making integration of new signals into adults’ perception a significant challenge. We believe that an approach informed by cognitive neuroscience is crucial for maximizing the true potential of new sensory technologies. Here, we present a framework for measuring and evaluating the extent to which new signals are integrated within existing structures of perception and experience. As our testbed, we use laboratory tasks in which healthy volunteers learn new, augmented perceptual-motor skills. We describe a suite of measures of (i) perceptual function (psychophysics), (ii) neural representations (fMRI/decoding), and (iii) subjective experience (qualitative interview/micro-phenomenology) targeted at testing hypotheses about how newly learned signals become integrated within perception and experience. As proof of concept, we provide example data showing how this approach allows us to measure changes in perception, neural processing, and subjective experience. We argue that this framework, in concert with targeted approaches to optimizing training and learning, provides the tools needed to develop and optimize new approaches to human sensory augmentation and substitution.

## Introduction

Wearable devices and technologies provide unprecedented scope to augment or substitute perceptual abilities. For example, devices can translate distance to auditory or tactile signals [1–3] to improve navigation for visually impaired people or convey signals not normally perceptible, such as magnetic North [4] or electromagnetic radiation [5].

A crucial bottleneck in abilities to make effective use of new signals is the sensory processing architecture of the human brain. A key insight from cognitive neuroscience is that perception and decision-making take place across many levels [6]. A simplified account would highlight, on the one hand, low-level “sensory” areas, where information processing is largely fast, bottom-up, and automatic and, on the other hand, higher-level “decision” areas, where processing is more effortful, goal-directed, and explicit. During skill acquisition, people proceed from a more deliberate and effortful approach to one that is more automatic [7, 8]—consider e.g. learning to drive or to play an instrument.

When it comes to perceptual skills, the most automatic or natural skill use would be expected to involve reorganization of basic sensory processing. Major reorganization of this kind is seen in individuals whose experience has been atypical from birth or very early life—e.g. when brain areas usually associated with vision carry out auditory processing of spatial information [9]. There is substantial potential for the brain to reorganize in this way in early life [10], but less is known about the potential for adult or lifelong learning to use alternative signals for perception.

New perceptual skills could be supported by a spectrum of mechanisms—from reorganization of low-level sensory networks to more explicit or deliberate strategies on the other. These are likely to have very different implications for the user’s functional abilities, ease of use, and subjective experience. We argue, therefore, that to optimize use and adoption of new technologies to enhance perception, it is crucial to integrate perspectives from across the cognitive sciences to understand at which level a new skill is implemented. This understanding will allow us to develop and optimize tools and technologies that can support and augment human perception in the most effective and engaging ways.

## Tasks and Approaches

### Overview

**Fig. 1:**
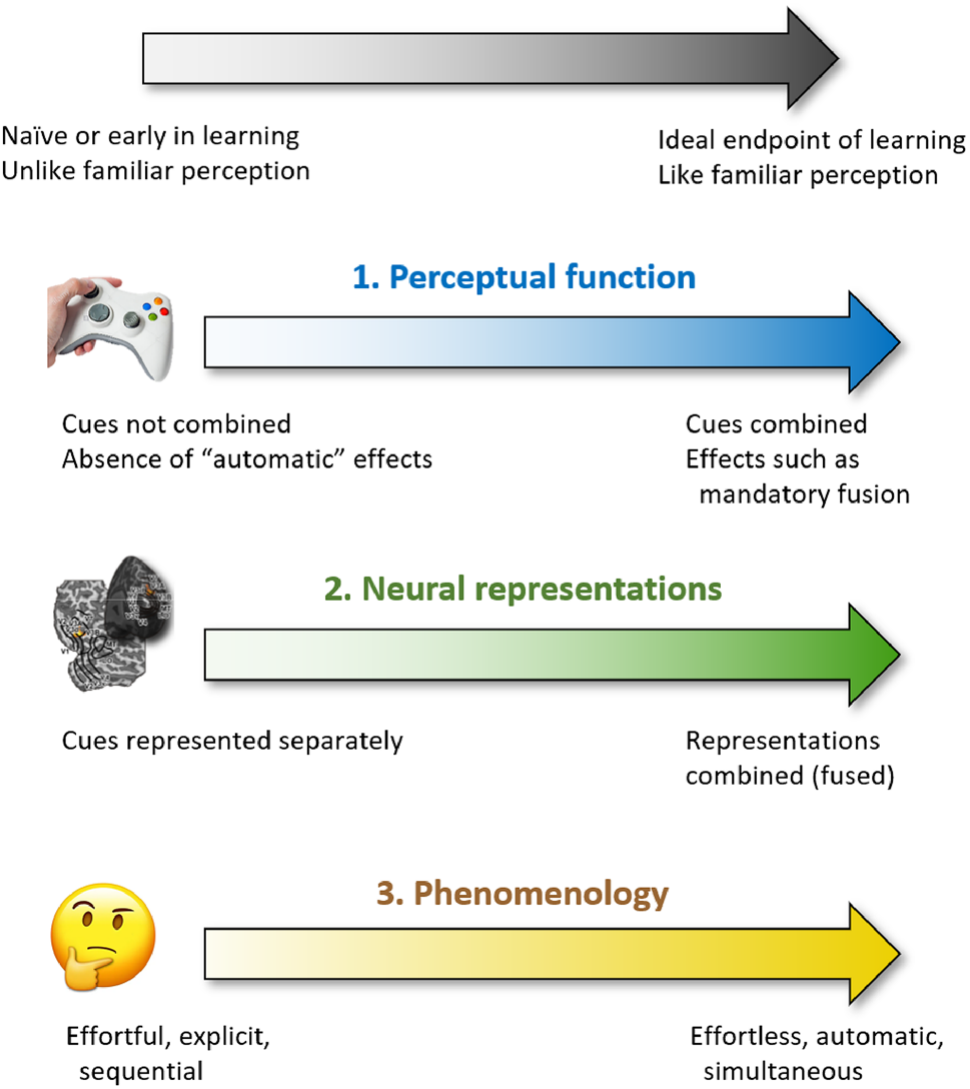
A framework for evaluating hypothesized changes in integration of a new signal within perception along three dimensions: perceptual function, neural representations, and phenomenology

**Fig. 2:**
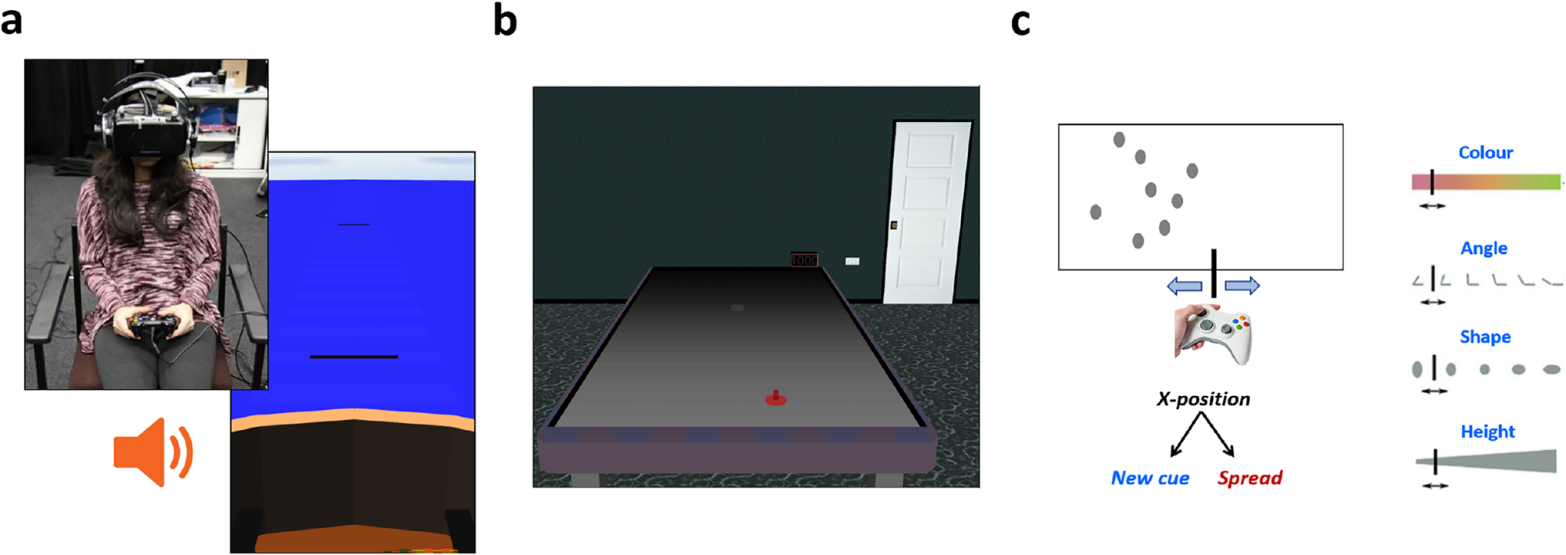
Example laboratory studies, training use of a new audio cue to judge **a**. target positions [14, 15] and to **b**. intercept moving targets [16], in VR. **c**. Desktop task for learning new visual cues to left–right position [17]

We present a framework (Fig. 1) for evaluating the extent to which new signals are integrated within existing structures of perception and experience. As our testbed, we use laboratory tasks in which healthy volunteers learn new, augmented perceptual-motor skills (Fig. 2). We outline a suite of measures of (i) perceptual function, (ii) neural representations, and (iii) subjective experience, targeted at testing hypotheses about the manner in which new signals become integrated within perception and experience. In each case, we outline why the approach provides crucial information and provide examples of its implementation.

### Laboratory Tasks

To evaluate integration of novel sensory signals, laboratory tasks should provide a controlled and repeatable way to elicit judgments or actions based on a novel sensory signal and/or familiar signals. Many of our tasks are designed in the manner of classic cue combination experiments [11], in which two independent information sources redundantly signal the same property in the world. This approach tests how different signals are *integrated* within perceptual judgements. In such studies, people may e.g. judge the sizes of objects using vision and touch [12], or judge the location of targets using vision and audition [13]. This approach has been applied to sensory augmentation/substitution by, for example, providing a new auditory cue to spatial position in VR (Fig. 2a, b) [14–16], or a new visual cue to left–right location in a desktop task (Fig. 2c) [17].

### Perceptual Function: Psychophysics

Classic methods from psychophysics [18] and particularly cue combination [11] can be used to measure gains in perceptual function when using a new signal. This is crucial for judging its practical effectiveness. Studies that also provide insight into the underlying computations are crucial steps towards a mechanistic understanding of how the signal operates. This allows for a better explanation of why certain approaches work, while others fail, for predictions about the most effective coding principles to convey novel information, and for interpretation of the functional results in relation to the other analyses described below.

In cue combination experiments [11], new and familiar cues are presented separately and together to measure how they interact, comparing human responses with predictions of different information processing models. Using this approach, studies of sensory augmentation have shown that (i) a new signal is rapidly combined with existing signals to improve precision [14, 15, 17]; (ii) signals are reweighted flexibly as they change in reliabilities [14]; (iii) however, this behaviour can fall short of the “statistically optimal” combination [14, 15, 17] that is common in multisensory perception with naturalistic signals [12, 13], and (iv) need not lead to “mandatory” (impossible to over-ride) fusion [15, 19], as perception with highly familiar signals can [20]. However, with the right coding scheme, cues can become partly automatic [4, 21, 22].

This approach provides important information about how effectively, and by which algorithms, a new signal participates in perception and decision-making. A prediction (Fig. 1) is that with extended and optimized training regimes, a new signal comes to behave like familiar signals on psychophysical measures of combination.

### Neural Representations

Neuroimaging methods such as fMRI, EEG, and MEG can reveal neural representations of sensory signals. For example, familiar visual and auditory signals to spatial location are processed across a hierarchy ranging from low-level primary “sensory” areas to higher-level secondary and “decision” areas [23, 24]. A new challenge is tracing the neural representations of newly learned, augmented signals [4, 25].

A promising approach to evaluating the degree to which a new signal is integrated within neural representations, for example in low-level “sensory” areas, is using information decoding (fMRI with multi-voxel pattern analysis) to determine which neuronal populations are combining (averaging) them into a single estimate. This approach has shown fusion of familiar, visual depth cues in visual cortex [26] and emergence of this in human development accompanying perceptual abilities to combine depth cues [27].

**Fig. 3:**
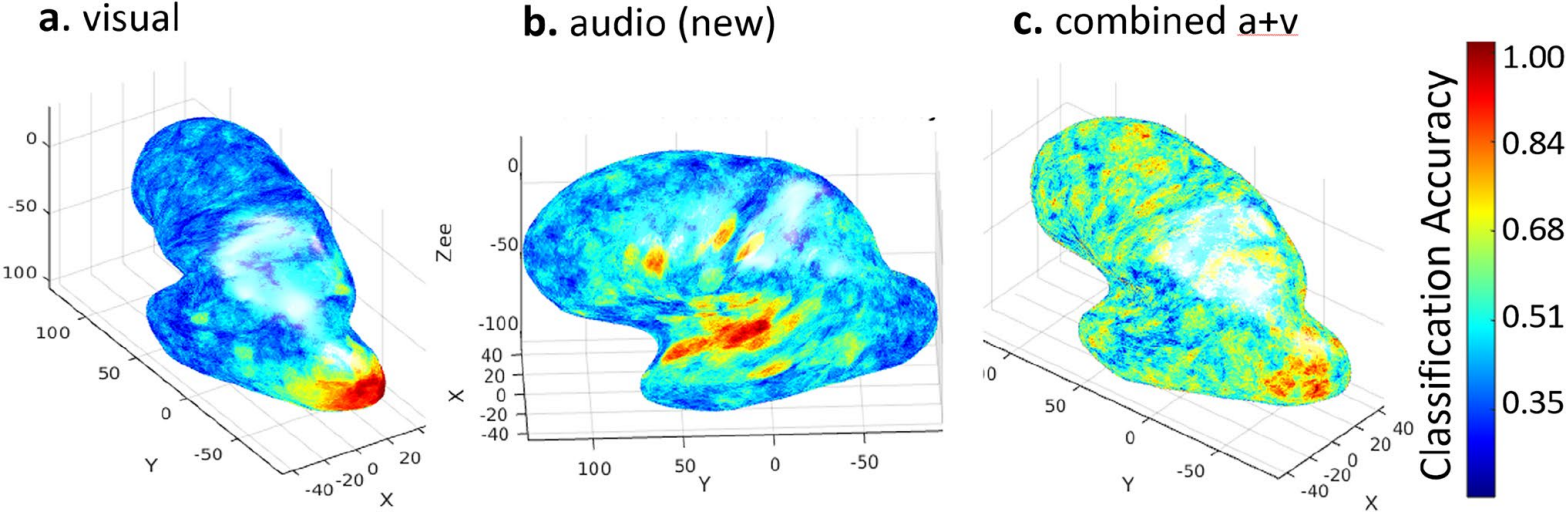
Single hemisphere (left) visualization of decoding accuracy on inflated brain surface for a single participant’s neural representations of **a**. visual depth, highlighting primary visual regions, **b**. a novel auditory cue, with reliable classification in primary auditory cortices, and **c**. combination of these, with classification extending into secondary areas

Current proof-of-concept work (Fig. 3) shows that e.g. both visual (familiar) and auditory (novel) cues can be decoded from within primary cortical areas (A and B) and decoding of combined cues can be achieved in regions extending up the visual hierarchy into secondary areas (C). These analyses have been developed to offer reliable decoding on a single-participant level through a deep-data approach, paving the way for longitudinal studies of the neural representation of depth as a new (e.g. audio) cue is learned.

### Phenomenology: Experiential measures

**Fig. 4:**
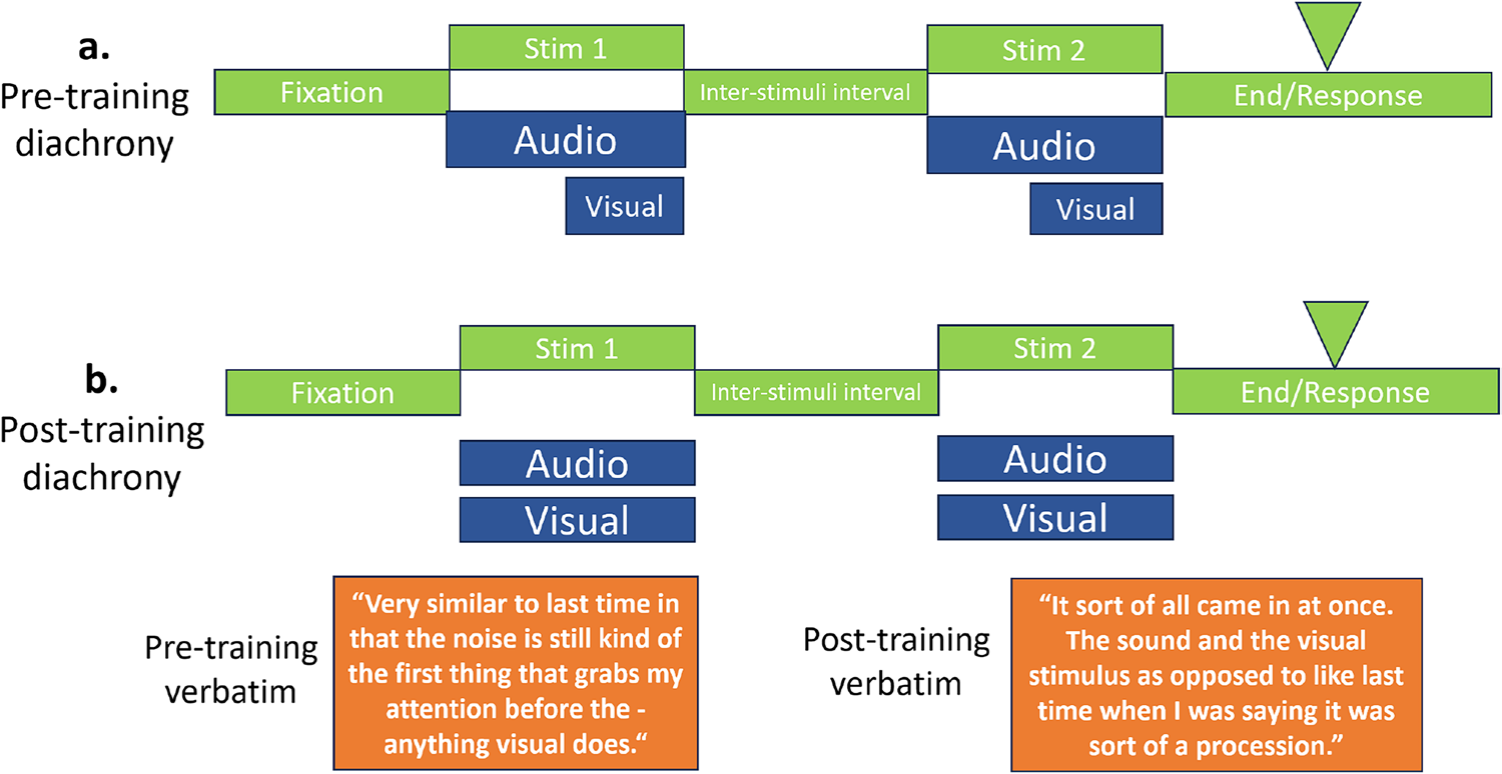
Depiction of a specific diachronic structure from a micro-phenomenological interview applied to an audio-visual depth task. Green boxes represent the experimental time course, blue boxes correspond to dimensions of the experience, and when they are reported to have arisen relative to stimuli, example key verbatim excerpts are in orange boxes

To gain a more complete understanding of sensory augmentation, we need to assess what it is like, subjectively, to sense in a new way. That is, investigations should also engage with the phenomenology of perceiving with novel sensory information. A technique that enables experimental contact with experience is Micro-phenomenology [28]. Employing this technique, we can observe the subjective changes accompanying training with, and exposure to, new sensory cues. For example, our initial data suggest when two cues (familiar and novel) are simultaneously presented, they are often initially subjectively perceived sequentially, with participants attending to one and then the other (Fig. 4a). After a period of training, the subjective temporal dynamics of this perception can change, such that the different sensory cues merge and are experienced together (Fig. 4b). Together with function (2.2) and neural representations (2.3), this approach provides a crucial third perspective on how perception and experience are changed through extended use of a new sensory device.

## Conclusions

There are exciting prospects ahead for augmenting human perceptual abilities. We have argued that evaluating and developing such approaches in the context of the organization of sensory information processing in the brain is crucial for making them effective. We have outlined three sets of tools for experimentation and analysis, which inform one another and together comprise a cognitive neuroscience framework for evaluating integration of new signals within perception (2.3), brain representations (2.4), and subjective experience (2.5).

Current work using this framework is allowing us, for example, to start to match qualitative findings of changes in subjective experience to functional and neuronal changes. This triangulation of evidence from different sources is beginning to uncover a route by which we can learn to use new sensory information that speaks to function, underlying neurophysiology, and subjective experience [28, 29] (Fig. 1). This combination of techniques is, however, in its infancy. Therefore, challenges abound, such as how to integrate data with different structures into a coherent representation. For example, micro-phenomenological descriptions are most accurate when referring to specific instances of experience, while neuroimaging measures tend to average brain activity over longer durations.

Our framework can assess the degree of sensory integration of new signals, but this in turn depends, of course, on the chosen devices, signals, and training regimes. For example, sensory augmentation in disability can be reciprocally optimized. By capturing how individuals function with (psychophysics) and experience (phenomenology) device use and relate it to the underlying physiology (e.g. with neuroimaging), it is possible to make modifications that are both scientifically motivated and experientially appreciated by the individual user. Our proposal is that optimizing these should be done in the context of the present cognitive neuroscience framework for understanding how new signals are processed and experienced.

## Data Availability

This is an opinion/review article—there are no data associated with this paper.
